# An Epidemic of Parvovirus B19-Induced Aplastic Crises in Pediatric Patients with Hereditary Spherocytosis Following the COVID-19 Pandemic: A Single-Center Retrospective Study

**DOI:** 10.3390/children12060772

**Published:** 2025-06-13

**Authors:** Paola Giordano, Valentina Palladino, Giuseppe Lassandro, Stella Spina, Giovanni Carlo Del Vecchio

**Affiliations:** Pediatric Unit “B. Trambusti”, Interdisciplinary Department of Medicine, University of Bari Aldo Moro, 70121 Bari, Italy; paola.giordano@uniba.it (P.G.); valentina.palladino@policlinico.ba.it (V.P.); giuseppe.lassandro@uniba.it (G.L.); s.spina6@studenti.uniba.it (S.S.)

**Keywords:** aplastic crisis, parvovirus B19, spherocytosis, COVID-19

## Abstract

**Background:** Parvovirus B19 is the major cause of transient aplastic crisis in children with hereditary spherocytosis (HS) inhibiting erythropoiesis and leading to a severe drop in hemoglobin levels, requiring hospitalization and transfusional support. During the COVID-19 pandemic, the circulation of non-COVID respiratory viruses, such as parvovirus B19, initially declined but subsequently increased abruptly following the relaxation of containment strategies. Moreover, it remains unclear whether this has resulted in a rise in parvovirus B19-induced aplastic crises among individuals with HS. **Methods:** This retrospective, single-center study conducted at the Pediatric University Hospital of Bari (Italy) aims to describe the clinical characteristics and frequency of parvovirus B19-induced aplastic crises in pediatric patients with HS before and after the COVID-19 Public Health Emergency of International Concern (PHEIC, 30 January 2020–5 May 2023). The study was divided into four distinct periods: Period A: from 1 December 2018 to 31 December 2019, representing one year before the declaration of the PHEIC; Period B: from 1 June 2023 to 30 June 2024, representing one year after the cessation of the PHEIC; Period C: before 1 December 2018; Period D: from 1 January 2020 to 31 May 2023, which refers to the pandemic period. **Results:** A total of 30 patients (55% of the study population, *n* = 55) experienced a parvovirus B19-induced aplastic crisis. The frequency of these crises in Period B was significantly higher than in Period A (*p* < 0.0001). **Conclusions:** This study suggests a substantial increase in parvovirus B19-induced aplastic crises among children with HS following the COVID-19 outbreak indicating a potential impact of public health containment strategies on parvovirus B19 infection rates.

## 1. Introduction

Hereditary spherocytosis (HS) is the most common cause of inherited hemolytic anemia due to a defect in erythrocyte membrane proteins (e.g., spectrin, ankyrin, band 3 and band 4.2 proteins), resulting in spherical red cells with reduced deformability and premature destruction in the spleen. HS is inherited in an autosomal dominant pattern in approximately 75% of cases, although autosomal recessive inheritance and de novo mutations have also been reported [1,2]. Mutations in the *ANK1*** ** gene, which encodes ankyrin-1, and in the *SPTB* gene, which encodes β-spectrin, are reported in 50–60% and 15–30% of autosomal dominant cases, respectively. Mutations in the *SPTA1* gene (encoding α-spectrin) and *EPB42* gene (encoding protein 4.2) are rarer and generally follow an autosomal recessive inheritance pattern. Changes in *SLC4A1*, the gene encoding band 3, are found in approximately 15% of cases and are inherited in an autosomal dominant manner [3].  

HS occurs in about 1 in 2000 individuals [1]. The clinical severity of HS ranges from asymptomatic forms to severe anemia requiring blood transfusions. The diagnosis of HS is primarily based on family history, typical clinical symptoms (such as jaundice, splenomegaly, and pallor), and laboratory findings (anemia, spherocytes on blood smear, elevated MCHC and reticulocytosis), without the need for additional testing. In doubtful cases, screening tests such as the eosin-5′-maleimide (EMA) binding test, cryo-hemolysis, or erythrocyte membrane electrophoresis (SDS-PAGE) can be performed, while genetic analysis is generally unnecessary for diagnosis. The major complications of HS include gallstones, hemolytic episodes, and aplastic crises [4,5].  

The major complications of HS include chronic hemolytic anemia, gallstones, splenomegaly, increased susceptibility to infections (such as post-splenectomy sepsis), and hematologic crises, including hemolytic, aplastic, and rarely megaloblastic crises [4,5,6,7,8,9,10,11,12].  

Parvovirus B19 is the primary cause of transient aplastic crisis in children with HS. Parvovirus B19 is a single-stranded, non-enveloped DNA virus belonging to the Parvoviridae family, responsible for erythema infectiosum, a common self-limited rash illness in children [13,14,15,16]. However, parvovirus B19 targets erythroid progenitor cells in the bone marrow, inhibiting erythropoiesis and causing transient erythroblastopenia and reticulocytopenia, which are generally not clinically significant in healthy subjects [17].  

In patients with HS, who already have a shortened red blood cell lifespan, this results in a transient aplastic crisis, causing a severe drop in hemoglobin, and requiring hospitalization [18]. The crisis is usually self-limited and resolves within two weeks. However, supportive care, including red blood cell transfusions, may be necessary to manage the condition [19].  

Although aplastic crisis is a well-recognized complication of HS, the real incidence of aplastic crises in patients with spherocytosis is unknown. Furthermore, prior to the COVID-19 pandemic, published cases were limited and mainly consisted of sporadic reports or small case series [13,16,20].

During the COVID-19 pandemic, health and social measures were implemented worldwide to limit the spread of the virus. COVID-19 is an airborne virus that is primarily transmitted between people through close contact and exposure to respiratory droplets. To prevent transmission, widespread use of personal protective equipment, social distancing, and hand hygiene were adopted. While these measures were aimed at controlling COVID-19, they also contributed to a significant reduction in the spread of other viral agents (e.g., influenza virus, rhinovirus, respiratory syncytial virus, parvovirus B19) [21,22]. Subsequently, the removal of these safety measures led to an increase in the spread of viral agents and the incidence of clinically evident infections [22,23].

After the COVID-19 pandemic, several European countries reported unusually high numbers of human parvovirus B19 infections, with potential implications for vulnerable individuals, such as pregnant women and patients with underlying hematologic conditions [24,25].

Although an increase in parvovirus B19 infections has been described after the COVID-19 pandemic, currently no studies have reported whether the relaxation of containment measures after the pandemic led to an increase in the frequency of parvovirus B19-induced aplastic crises among patients with HS.

Here, we report an epidemic of parvovirus B19-induced aplastic crises following the COVID-19 pandemic in a single-center series of pediatric patients with HS, describing both their clinical characteristics and frequency. In our study, we compared the frequency of aplastic crises before and after the pandemic, with the aim of providing new epidemiological data on parvovirus B19-induced aplastic crises in patients with HS in the post-pandemic era.

## 2. Materials and Methods

This retrospective, single-center study, conducted at the Pediatric University Hospital of Bari (Bari, Italy), aims to describe the clinical characteristics and frequency of parvovirus B19-induced aplastic crises in pediatric patients with hereditary spherocytosis before and after the COVID-19 Public Health Emergency of International Concern (PHEIC, 30 January 2020–5 May 2023).

Inclusion criteria were the following:

• Age between 0 and 17 years and 11 months at the end of enrollment period (30 June 2024), 
• Diagnosis of HS according to the British Journal of Haematology  guidelines for the diagnosis and management of HS [4], 
• Patients with regular follow-up at our center, defined as annual clinical, laboratory, and abdominal ultrasound assessments, from the time of diagnosis until the end of the enrollment period,
• Availability of complete clinical, laboratory, and virological data as described below. 

**Exclusion criteria** included the following: 

• History of splenectomy prior to parvovirus B19 infection, 
• Patients with incomplete clinical, laboratory, or virological data were excluded to ensure data accuracy and reliable analysis. Although excluding incomplete records can introduce selection bias, potentially affecting internal validity and generalizability, in this study, no patients were excluded from the study. This is likely due to the single-center design and the limited, well-controlled patient cohort, which ensured complete clinical, laboratory, and virological documentation. 

**Figure 1:**
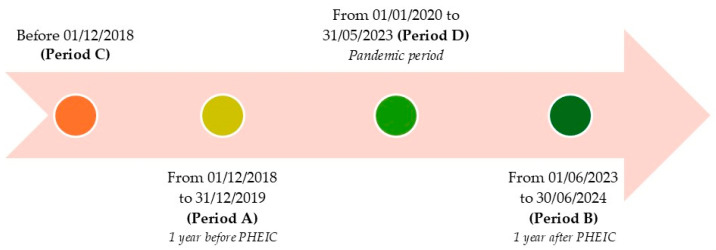
Study timeline and definition of observation periods.

The study  was divided into four distinct periods (Figure 1): 

• **Period A:** from 1 December 2018 to 31 December 2019, representing one year before the declaration of the PHEIC; 
• **Period B:** from 1 June 2023 to 30 June 2024, representing one year after the cessation of the PHEIC; 
• **Period C:** all parvovirus B19-induced aplastic crises recorded before 1 December 2018; 
• **Period D:** from 1 January 2020 to 31 May 2023, which refers to the pandemic period. 

The entire month of January 2020 was included in Period D for practical reasons to avoid splitting data within a single month and to maintain clarity and consistency in temporal categorization. Notably, no cases of parvovirus B19-induced aplastic crisis were recorded during January 2020. Therefore, this inclusion did not impact the internal validity of the temporal analysis.  

The comparison of the frequency of parvovirus B19-induced aplastic crises between periods A and B was based on the number of total susceptible cases, excluding patients previously immunized.

Demographic and clinical data were collected for all patients, including sex, age at the end of enrollment period (in months), and age at diagnosis of HS (in months). Patients were diagnosed and clinically classified as having ‘mild’, ‘moderate’, or ‘severe’ forms of HS according to *the British Journal of Haematology* guidelines for the diagnosis and management of HS [4]. Additional information regarding cholecystectomy and/or splenectomy was collected.

Regarding parvovirus B19-induced aplastic crisis, detailed information was recorded, including the presence of clinical symptoms (such as asthenia, fever, spleen enlargement, rash at onset or during follow-up) and hematological parameters at onset, including hemoglobin, absolute and percentage reticulocyte count, neutrophil count, and platelet count.

Virological markers for parvovirus B19, Human Herpes Simplex virus (HSV 1-2), Epstein–Barr virus (EBV), and Cytomegalovirus (CMV) were evaluated by real-time PCR and/or serological testing (specific IgM antibodies).

Time to return to a steady state for each hematological parameter was measured from the first laboratory test at hospital admission and defined as the number of days required to return to the patient’s baseline interictal levels as documented during previous follow-up visits.

Transfusion requirements were assessed for each patient with parvovirus B19-induced aplastic crisis, including the number of leukocyte-depleted red blood cell units transfused and the total transfused volume (ml/kg). Additional complications, such as splenic infarction, as well as the recurrence of anemia and/or reticulocytopenia and/or the need for further transfusions during the 6-month follow-up period, were also recorded.

Reticulocytopenia was defined as a reticulocyte count of less than 50,000/mm^3^ [26]. Bicytopenia was defined as a reduction in any two blood cell lines, and pancytopenia as a reduction in all cell lines (erythrocytes, leukocytes, and platelets) [27]. An increase in splenomegaly was defined as an ultrasound and clinically measured spleen enlargement of 2 cm or more.  

### Statistical Analysis

The Stat View program (Abacus Concepts, Berkeley, CA, USA) was used for statistical analysis. Data were expressed as percentages (referring to either the entire sample or subgroups) and as medians with ranges (minimum and maximum values). Comparisons between groups were performed using the Chi-square test. *p* values < 0.05 were considered statistically significant.

## 3. Results

The characteristics of the study population are reported in Table 1.

A total of 30 patients (55% of study population, *n* = 55) experienced parvovirus B19-induced aplastic crisis. The crises involved 22 males (73%) and 8 females (27%) with a median age of 78 months (range: 9–196 months).

The median age at diagnosis of HS was 20 months (range: 2–172 months) while the median age at the end of the enrollment period was 147 months (range: 10–215 months). Regarding the clinical forms of HS, 13 patients (43%) were classified as mild, 14 (47%) as moderate, and 3 (10%) as severe. Cholelithiasis was documented in 13 patients (43%), and 11 (37%) had undergone cholecystectomy. Although 4 patients (13%) had undergone splenectomies, none of these procedures occurred before the onset of the aplastic crisis.

Parvovirus B19-induced aplastic crises occurred during the periods reported in Table 2. A total of 4 patients (13%) developed parvovirus B19-induced aplastic crisis in Period A, 17 patients (57%) in Period B, and 9 patients (30%) in Period C. No patients developed aplastic crisis in Period D. These data are reported in Table 2.

**Table 2:** Patients with parvovirus B19-induced aplastic crisis recorded in the studied population.

Total Number of Patients with Parvovirus B19-Induced Aplastic Crisis		
*n*	30	
Patients with aplastic crisis in period A *, *n* (%) **	4	(13%)
Patients with aplastic crisis in period B *, *n* (%) **	17	(57%)
Patients with aplastic crisis in period C *, *n* (%) **	9	(30%)
Patients with aplastic crisis in period D *, *n* (%) **	0	(0%)

* Period A: from 1 December 2018 to 31 December 2019; Period B: from 1 June 2023 to 30 June 2024; Period C: all aplastic crisis cases recorded before 1 December 2018; Period D: from 1 January 2020 to 31 May 2023. ** Percentages refer to the total number of patients with parvovirus B19-induced aplastic crisis (*n* = 30).

The frequency of parvovirus B19-induced aplastic crises was significantly higher in Period B (17 of 30 patients with aplastic crisis, 57%) than in Period A (4 of 30 patients with aplastic crisis, 13%), *p* < 0.0001. Data are presented in Table 3.

**Figure 2:**
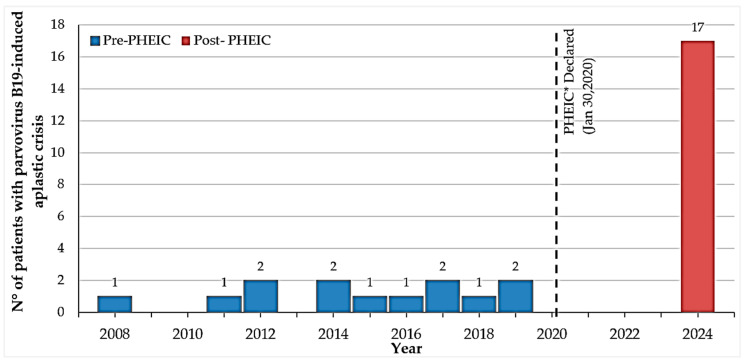
Annual incidence of parvovirus B19-induced aplastic crises among children with hereditary spherocytosis. * PHEIC = COVID-19 Public Health Emergency of International Concern (30 January 2020–5 May 2023).

Figure 2 illustrates the annual incidence of parvovirus B19-induced aplastic crises among children with HS across the four defined study periods.

All observed cases of aplastic crisis (*n* = 30) required urgent hospitalization. Among patients who developed a parvovirus B19-induced aplastic crisis, the most common presenting symptoms were asthenia and fever, reported in 100% and 87% of cases, respectively. A total of 43% of patients with aplastic crisis presented with increased spleen size, while the characteristic parvovirus B19 rash was observed in a minority of patients (3% at crisis onset and 13% during follow-up). Clinical symptoms are reported in Table 4.  

Virological investigations showed that all patients with aplastic crisis (*n* = 30) were positive for parvovirus B19 IgM. In all patients, the infection was confirmed by viral load detection using real-time PCR. The median viral load was 26,974,072 copies/mL (range: 4,910,441–50,000,000 copies/mL). Additionally, HSV1-2, HHV-8, CMV, and EBV IgM positivity was detected in 57%, 53%, and 60% of patients with parvovirus B 19-induced aplastic crisis, respectively. In almost all cases, real-time PCR did not confirm the presence of these viruses, except for 4 patients (13%) of those with aplastic crisis who had a concurrent co-infection with EBV.

In all subjects with aplastic crisis (*n* = 30), a decrease in baseline interictal hemoglobin and reticulocyte values was recorded. Moreover, neutropenia and thrombocytopenia were recorded in 63% and 67% of cases, respectively. Overall, 30% of patients presented with bicytopenia, while 50% developed pancytopenia (Table 5).

During the aplastic crisis, all patients (*n* = 30) developed severe hematological abnormalities with a median hemoglobin value of 6.1 g/dL (range: 2.4–8.4 g/dL), reticulocyte percentage of 0.4% (range: 0.1–2.1%), and absolute reticulocyte count of 9900/mm^3^ (range: 2232–30,000/mm^3^). Additionally, the median platelet and neutrophil counts were 101,000/mm^3^ (range: 30,000–511,000/mm^3^) and 1315/mm^3^ (range: 371–21,318/mm^3^), respectively. Median hematological values (range: min–max) are reported in Table 6.

Hemoglobin and reticulocyte levels remained below baseline for a median of 30 days (range: 6–59 days) and 9 days (range: 3–19 days), while neutropenia and thrombocytopenia persisted for a median of 5 days (range: 1–12 days) and 6 days (range: 2–19 days), respectively. The median duration of hematological parameters alterations (range: min–max) is reported in Table 7.

All patients with aplastic crisis (*n* = 30) required at least one leukocyte-depleted red blood cell transfusion (median: 2, range: 1–5 transfusions). The median total volume of blood transfused per patient was 20 mL/Kg (range: 8–40 mL/Kg). One of the thirty patients with aplastic crisis (3%), who had a co-infection with EBV, developed a splenic infarction.

During the 6 months following the crisis, 6 out of 30 patients with aplastic crisis (20%) experienced a further decline in interictal hemoglobin levels, requiring additional transfusions. These episodes were accompanied by a recurrence of reticulocytopenia.

## 4. Discussion

The COVID-19 pandemic represented an unprecedented global health emergency, causing a significant increase in mortality and morbidity worldwide. In the early stages of the pandemic, containment strategies and the widespread use of personal protective equipment contributed to reducing the spread of COVID-19 prior to the availability of vaccines and specific therapies [28]. Moreover, the implementation of non-pharmaceutical interventions (NPIs) also appeared to drastically reduce the circulation of other respiratory seasonal viruses such as influenza virus, respiratory syncytial virus, parvovirus B19, as well as bacterial pathogens like Streptococcus pneumoniae and Streptococcus pyogenes [29,30,31,32]. However, when containment strategies were relaxed, a rebound in the spread of numerous non-COVID-19 pathogens was observed [33,34,35].

Several studies have reported a significant rise in parvovirus B19 infections after the pandemic, with potential consequences for vulnerable individuals, such as pregnant women, blood transfusion recipients, and patients with hematological hemolytic disorders who are susceptible to bone marrow suppression leading to aplastic crisis [24,25,36,37]. A recent review reported that the incidence rate of parvovirus B19-induced aplastic crises in pediatric patients with sickle cell disease was 3.6 times higher in 2024 compared with the overall rate during the 2010–2023 period [38].

Although an increase in parvovirus B19 infections has been described after the pandemic [24,25,36,37], it remains unclear whether there has been a corresponding rise in aplastic crises among patients with HS.

The present study describes an unusual epidemic of parvovirus B19-induced aplastic crises following the COVID-19 pandemic in a single-center cohort of pediatric patients with HS. According to previous studies, reporting a reduction in the circulation of non-COVID viruses during the pandemic [29,30,31], no cases of aplastic crisis were recorded during the PHEIC. Furthermore, we compared the frequency of parvovirus B19-induced aplastic crises before and after the PHEIC and found a statistically significant increase in the post-pandemic period. Specifically, the number of children hospitalized for parvovirus B19-induced aplastic crisis between June 2023 and June 2024 was 4.25 times higher than during the 2018–2019 period.

Before the COVID-19 pandemic, parvovirus B19-induced aplastic crisis in subjects with HS appeared to have a limited frequency, as evidenced by the fact that most of the available literature consisted of isolated case reports or small case series [13,14,19,20]. Several studies have described the clinical course, hematological findings, and complications of parvovirus B19-induced aplastic crisis in patients with HS, highlighting the need for urgent hospitalization, medical management, and transfusion support [13,14,16,19,20,39,40]. According to previous studies [13,14,16,19,20], we found that the most common symptoms among patients with parvovirus B19-induced aplastic crisis were asthenia and fever. Furthermore, a decrease in hemoglobin and reticulocyte levels was observed in all patients, requiring urgent hospitalization and transfusion support. Most patients presented with bi- or trilineage cytopenia that resolved spontaneously within a few weeks. However, during the 6 months following the crisis, some patients experienced a further decline in interictal hemoglobin levels, requiring additional blood transfusions, with a recurrence of reticulocytopenia. As a complication, one patient with EBV co-infection developed splenic infarction.

Our study confirms that parvovirus B19-induced aplastic crisis is a severe complication in patients with HS, requiring hospitalization, transfusion support, and exposing patients to potential further complications. Moreover, our study is the first to report a post-pandemic abrupt increase in parvovirus B19-induced aplastic crises among pediatric patients with hereditary spherocytosis.

The abrupt increase in parvovirus B19-induced aplastic crises can be explained by the concept of “immune debt” [41]. During the COVID-19 pandemic, the reduced circulation of infectious agents may have led to inadequate pathogen-induced immune responses, including a decline in herd immunity and in the production of pathogen-specific antibodies. Furthermore, this has resulted in a decreased maternal passive immunity [42]. Consequently, a larger number of children became susceptible to infectious diseases, contributing to the workload on the healthcare system and leading to a rise in hospitalizations and medical treatments. The insufficient immune response may also affect immune system maturation and predispose to the development of allergic and autoimmune disorders [43].

Furthermore, the recent increase in respiratory infections caused by non-COVID viruses may also be attributable to the concept of “immune theft” [44]. Several studies suggest that COVID-19 infection can directly compromise the immune system, leading to prolonged dysfunction. During the acute phase of the disease, COVID-19 has been shown to significantly alter immune function, particularly affecting the T and NK cell compartments [45,46]. Specifically, reduced T and NK cell counts and elevated levels of proinflammatory cytokines such as IL-6 have been observed, especially in patients with severe disease. Alterations in the T, NK, and B cell compartments—as well as persistent inflammation, CD8+ T cell activation, and changes in CD19 receptor expression—may continue for more than six months after infection. Moreover, COVID-19 infection induces a more intense and longer-lasting cellular immune response than other respiratory viruses, even in mild or asymptomatic cases [45,46,47]. This prolonged immune dysregulation may increase susceptibility to secondary viral infections, such as parvovirus B19, and warrants further investigation in predisposed patient populations [44].

This study has several limitations. First, it is a single-center, retrospective study involving a relatively small cohort of pediatric patients with hereditary spherocytosis. Due to the limited sample size, our analysis was limited to comparing the frequency of parvovirus B19-induced aplastic crises before and after the COVID-19 pandemic, without assessing potential differences in clinical severity, transfusion requirements, or other clinical characteristics. Further multicenter studies involving a larger number of patients with HS are needed to confirm our findings and to evaluate whether the clinical presentation and severity of parvovirus B19-induced aplastic crises differ between the post- and pre-pandemic periods. Additionally, prospective studies would be useful to evaluate whether the increase in parvovirus B19-induced aplastic crises among children with HS following the COVID-19 outbreak represents a transient fluctuation phenomenon or persists over time.

## 5. Conclusions

In conclusion, this study suggests a substantial increase in parvovirus B19-induced aplastic crises among children with HS following the COVID-19 outbreak, indicating a potential impact of public health containment strategies on parvovirus B19 infection rates. These findings provide preliminary evidence regarding changes in post-pandemic epidemiology of parvovirus B19 infection and the possible effects on vulnerable patients, such as children with HS. Further studies are needed to validate these findings, clarify their clinical significance, determine whether the observed increase in parvovirus B19-induced aplastic crises post-pandemic is temporary or sustained, and assess potential changes in clinical severity.

## Figures and Tables

**Table 1:** Clinical characteristics of the study subjects.

**Total Number of Study Subjects**		
*n*	55	
**Gender,** *n* (%) *		
M	34	(62%)
F	21	(38%)
**Age at time of study,** months		
Median (range: min–max)	151	(8–215)
**Age at diagnosis of spherocytosis,** months		
Median (range: min–max)	24	(2–203)
**Clinical forms of spherocytosis,** *n* (%) *		
Mild	34	(62%)
Moderate	18	(33%)
Severe	3	(5%)
**Patients undergoing cholecystectomy,** *n* (%) *	12	(22%)
**Patients undergoing splenectomy,** *n* (%) *	4	(7%)

* Percentages refer to the total number of study subjects (*n* = 55).

**Table 3:** Frequency of parvovirus B19-induced aplastic crises before and after PHEIC *.

	Crisis, Yes	Crisis, No	Total
Period A	4	40	44
Period B	17	13	40
Chi-square value: *p <* 0.0001			

* PHEIC = COVID-19 Public Health Emergency of International Concern (30 January 2020–5 May 2023).

**Table 4:** Clinical symptoms in subjects with parvovirus B19-induced aplastic crisis.

**Total of Subjects with Parvovirus B19-Induced Aplastic Crisis,** *n*	30	
**Asthenia,** *n* (%) *	30	(100%)
**Fever,** *n* (%) *	26	(87%)
**Spleen enlargement,** *n* (%) *	13	(43%)
**Rash at crisis onset,** *n* (%) *	1	(3%)
**Rash during follow-up,** *n* (%) *	4	(13%)

* Percentages refer to the total number of patients with aplastic crisis (*n* = 30).

**Table 5:** Hematological findings of subjects with parvovirus B19-induced aplastic crisis.

	Subjects, *n*	Subjects, (%) *
Worsening of baseline anemia	30	(100%)
Reticulocytopenia	30	(100%)
Neutropenia	19	(63%)
Thrombocytopenia	20	(67%)
Bicytopenia	9	(30%)
Pancytopenia	15	(50%)

* Percentages refer to the total number of patients with parvovirus B19-induced aplastic crisis (*n* = 30).

**Table 6:** Hematological findings during parvovirus B19-induced aplastic crisis.

**Hemoglobin,** (g/dL)		
Median (range: min–max)	6.1	(2.4–8.4)
**Reticulocyte count,** (%)		
Median (range: min–max)	0.4	(0.1–2.1)
**Reticulocyte count,** (*n*/mm^3^)		
Median (range: min–max)	9900	(2232–30,000)
**Neutrophil count,** (*n*/mm^3^)		
Median (range: min–max)	1315	(371–21,318)
**Platelet count,** (*n*/mm^3^)		
Median (range: min–max)	101,000	(30,000–511,000)

**Table 7:** Duration of alteration of hematological parameters.

**Hemoglobin,** *n*° days		
Median (range: min–max)	30	(6–59)
**Neutrophil count,** *n*° days		
Median (range: min–max)	5	(1–12)
**Platelet count,** *n*° days		
Median (range: min–max)	6	(2–19)
**Reticulocyte count,** *n*° days		
Median (range: min–max)	9	(3–19)

## Data Availability

The data presented in this study are available on request from the corresponding author to maintain control over intellectual property rights and ensure that the information is not used improperly.

## Abbreviations

The following abbreviations are used in this manuscript:

PHEIC	Public Health Emergency of International Concern
HS	Hereditary spherocytosis
EMA	Eosin-5′-maleimide binding test, cryo-hemolysis
SDS-PAGE	Erythrocyte membrane electrophoresis
NPIs	Non-pharmaceutical interventions
